# Prognostic impact of *Candida* spp. isolation and early antifungal therapy in ICU patients with peritonitis: A multicenter cohort study

**DOI:** 10.1016/j.aicoj.2026.100140

**Published:** 2026-08-25

**Authors:** Mattéo Mauget, Charly Angebault, Melchior Bardoul, Pierre Fillâtre, Pierre Bouju, Agathe Delbove, Guillaume Rieul, Yannick Fedun, Renaud Prével, Jean-Pierre Gangneux, Yoann Launey, Florian Reizine

**Affiliations:** a Medical Intensive Care Unit, CHU de Rennes, 35000, Rennes, France; b Intensive Care Unit, Vannes Hospital Center, 56000 Vannes, France; c Univ-Rennes, Surgical Intensive Care Unit, CHU de Rennes, 35000, Rennes, France; d Intensive Care Unit, Saint-Brieuc Hospital Center, 22000 Saint-Brieuc, France; e Intensive Care Unit, Lorient Hospital Center, 56100 Lorient, France; f CHU Bordeaux, Medical Intensive Care Unit, F-33000, Bordeaux, France, Univ Bordeaux, Centre de Recherche Cardio-Thoracique Inserm U1045, F-33600 Pessac, France; g Centre National de Référence des Mycoses et Antifongiques LA-AspC Aspergilloses chroniques, European Excellence Center for Medical Mycology (ECMM-EC), Centre hospitalier Universitaire de Rennes, F-35000 Rennes, France; h Univ Rennes, CHU Rennes, Inserm, EHESP, Irset (Institut de Recherche en Santé Environnement Travail) - UMR_S 1085, F-35000 Rennes, France

**Keywords:** Peritonitis, ICU, *Candida* spp., Antifungal therapy

## Abstract

**Background:**

*Candida* spp. is frequently isolated in critically ill patients with peritonitis. Guidelines recommend empirical antifungal therapy in high-risk cases, but the clinical significance of *Candida* spp. isolation in peritoneal fluid and the benefit of early antifungal therapy remain uncertain.

**Methods:**

We conducted a retrospective multicenter cohort study including all consecutive adults admitted with peritonitis to the ICUs of four hospitals in Western France between 2020 and 2022. Risk factors for *Candida* spp. isolation in peritoneal fluid were assessed using multivariate logistic regression. Outcomes were compared according to *Candida* spp. isolation in peritoneal fluid and, among affected patients, according to early antifungal therapy. Survival up to day-90 after peritonitis onset were compared using Inverse Probability Treatment-weighted Cox proportional hazards models.

**Results:**

Among 378 patients, *Candida* spp. was isolated in 81 (21.4%). Independent risk factors included antimicrobial therapy within the last 3 months (adjusted OR 2.15, 95% CI 1.20–3.81; p = 0.009), malnutrition (adjusted OR 1.84, 95% CI 1.10–3.14; p = 0.022), upper gastrointestinal origin (adjusted OR 2.04, 95% CI 1.20–3.44; p = 0.008) and diffuse peritonitis (adjusted OR 1.75, 95% CI 1.01–3.13; p = 0.049). *Candida* spp. isolation in peritoneal fluid was not associated with 90-day mortality (weighted HR 1.00, 95% CI 0.62–1.59; p = 0.991) and clinical courses were similar between groups. Among patients with *Candida* spp. in peritoneal fluid isolation, early antifungal therapy was not associated with 90-day mortality (weighted HR 1.37, 95% CI 0.58–3.22; p = 0.470) or clinical outcomes.

**Conclusions:**

In ICU patients with peritonitis, *Candida* spp. isolation in peritoneal fluid was not associated with 90-day mortality, and early antifungal therapy did not improve 90-day survival among patient with isolated *Candida* spp. in peritoneal fluid.

## Highlights

• *Candida* spp. was isolated in nearly one-quarter of ICU peritonitis cases.
• Prior antibiotics, malnutrition, upper GI origin and diffuse peritonitis increased the risk of *Candida* spp. isolation.
• *Candida* spp. isolation was not associated with worse 90-day outcomes.
• Early antifungal therapy showed no survival benefit at day 90.

## Introduction

Peritonitis is a common condition encountered in Intensive Care Units (ICUs), defined as an inflammation of the peritoneum, most often resulting from disruption of the gastrointestinal (GI) tract and the spread of pathogens into the abdominal cavity [1]. This life-threatening condition is the second cause of sepsis and warrants prompt intervention, including appropriate anti-infective therapy and complete surgical or radiological drainage, to prevent significant morbidity and mortality [[1], [2], [3]]. Isolated microorganisms include a broad spectrum of pathogens, with *Enterobacterales*, streptococci and anaerobic bacteria being the most prevalent [4,5]. In addition, *Candida* species are frequently recovered, especially in critically ill patients [4].

In secondary peritonitis, the prognostic significance of intra-abdominal *Candida* spp. isolation remains controversial. Previous studies have reported conflicting results, depending on the setting of acquisition and the severity of illness, ranging from no impact to increased mortality in critically ill patients [[6], [7], [8], [9], [10], [11]]. Consequently, current guidelines provide inconsistent recommendations regarding empiric antifungal therapy, with some advocating early initiation in high-risk or severely ill patients [[12], [13], [14]], while others restrict antifungal therapy to microbiologically documented infections [15].

These differing strategies are largely informed by evidence from candidemia, in which delayed antifungal therapy has been consistently associated with worse outcomes [[16], [17], [18], [19], [20]]. However, extrapolating these findings to *Candida* spp*.* isolation in peritoneal fluid may be inappropriate. Recent consensus definitions from the FUNDICU consortium no longer consider *Candida* spp. recovered from peritoneal fluid after gastrointestinal or urogenital perforation as sufficient evidence of invasive candidiasis when prompt and effective source control is achieved [21]. In this context, *Candida* spp. recovery from peritoneal samples may represent transient contamination or colonization rather than true invasive infection. Hence, its clinical relevance remains uncertain, and whether antifungal therapy improves outcomes in this setting is still debated [7,10,11,[22], [23], [24], [25], [26], [27]].

Therefore, we aimed to investigate the association between intra-abdominal *Candida* spp. isolation and 90-day mortality in critically ill patients with peritonitis, and to explore the impact of early antifungal therapy in this population.

## Methods

### Study design

The MOPI Candida study was an ancillary analysis of a retrospective multicenter cohort study, previously published [28,29]. Briefly, all patients admitted to the Intensive Care Units (ICUs) of four French hospitals (one university-affiliated hospital and three general hospitals) and diagnosed with peritonitis between January 2020 and December 2022 were included. Patients were initially identified using hospital administrative databases based on predefined ICD-10 codes (**Supplementary Table** 1). Study protocol was approved by the French Ethics Committee for Research in Anaesthesia and Intensive Care (IRB 00010254-2023-017). The study complied with STROBE reporting guidelines for observational studies [30].

### Data collection and definitions

Peritonitis was defined as an intra-abdominal infection requiring surgical or radiological source control and associated with microbiological sampling. Setting of acquisition was categorized as community-acquired, occurring before hospital admission or within the first 48 h. Conversely, nosocomial peritonitis, occurring after 48 h of hospital admission, were classified as early-onset or late-onset peritonitis, whether they occurred within the first 7 days of hospital stay or not. Peritonitis occurring within 30 days after abdominal surgery was defined as postoperative. Clinical, microbiological, and therapeutic data were extracted from electronic medical records using a standardized case report form. Comorbidities included obesity (body-mass index > 30 kg/m²), malnutrition (albumin level at admission <30 g/L and/or BMI<20 kg/m^2^), diabetes mellitus, cirrhosis, chronic kidney failure (GFR<60 mL/min/m^2^), chronic heart disease and immunosuppression (active hematological malignancy, solid cancer, solid organ transplantation (SOT), autoimmune disease, or long-term corticosteroid or immunosuppressive therapy). Severity at ICU admission was assessed by the Simplified Acute Physiology Score II (SAPS II) [31]. Sepsis and septic shock were defined according to Sepsis-3 criteria [32].

*Candida* spp. isolation was defined based on culture positivity of peritoneal fluid. Of note, patient with documented candidemia were excluded from the main analysis. To assess robustness of our findings, we performed sensitivity analyses including patients with concomitant candidemia. *Candida* species (further defined as *albicans* or non-*albicans*), direct examination positivity for yeasts of peritoneal fluid and azole resistances were recorded. Notably, all *C.glabrata* strains were classified Fluconazole-resistant, according to EUCAST guidelines. Early antifungal therapy was defined as antifungals administered within 48 hours after the index surgery, including whether echinocandins or fluconazole, provided that subsequent isolated *Candida* strain was sensitive to the drug given empirically. Notably, there was no standardized protocol across or within institutions regarding fungal workup and management of antifungals. Decisions were left to physicians in charge, that were encouraged to follow French national guidelines [12]. Patients were followed until day 90 after peritonitis onset or hospital discharge, whichever occurred first, with censoring of survivors at day 90.

### Objectives

The main objective was to compare 90-day all-cause mortality after peritonitis onset, according to *Candida* spp. isolation in peritoneal fluid.

Secondary objectives included the assessment of patients’ baseline characteristics and clinical courses, including source control achievement, ICU or hospital length of stay and ICU or hospital mortality, according to *Candida* spp. isolation. Last, we aimed to assess risk factors for *Candida* spp. isolation in peritoneal fluid among patients with peritonitis.

Among patients with isolated *Candida* spp., we also aimed to compare 90-day all-cause mortality and patient clinical courses after peritonitis onset, according to early antifungal therapy initiation. Last, we aimed to compared 90-day all-cause mortality after peritonitis onset according to early antifungal therapy in several subgroups, including patients with nosocomial peritonitis, septic shock, yeast-positive direct examination or pure *Candida* spp. cultures.

### Statistical analysis

Continuous data are reported as medians [Q1–Q3] and categorical variables as counts (percentages). Comparative analyses between groups were conducted using Mann-Whitney *U* test or Student's *t*-test for continuous variables depending on their distribution, and Chi² test or Fisher's exact test for categorical variables, as appropriate. Survival curves were estimated using the Kaplan-Meier method and compared between groups using log-rank tests.

To address potential confounding due to baseline imbalances between patients with and without *Candida* spp. isolation in peritoneal fluid, we performed a stabilized inverse probability of treatment weighting (IPTW) approach (Model 1) [33]. We first estimated non-parsimonious propensity scores (PS) using multivariate logistic regression incorporating all patient baseline characteristics with at least 5 patients per group (**Supplementary Table** 2). Stabilized weights were then calculated. Marginal IPTW probabilities (<5^th^ percentile or >95^th^ percentile) were winsorized to improve weighting stability [34].

Similarly, we performed a second model (Model 2) focusing on the effect of early antifungal therapy among patients with isolated *Candida* spp. in peritoneal fluid. We calculated stabilized weights and winsorised marginal IPTW probabilities.

Effectiveness of each weighting approach was systematically evaluated using absolute standardized mean differences (SMD) for each covariate before and after weighting, with values below 0.2 considered as adequate balance. To assess model robustness, we performed sensitivity analyses including bootstrap validation of the propensity score models (1000 iterations) and event per variable (EPV) calculations.

Primary outcome of 90-day all-cause mortality was analyzed using weighted Cox proportional hazards model, with results expressed as weighted Hazard Ratios (HR) with their corresponding 95% confidence intervals (CI), and survival probabilities were represented through weighted Kaplan-Meier curves.

Factors associated with *Candida* spp. isolation in peritoneal fluid were identified using univariate then multivariate logistic regression with backward stepwise selection (entry p < 0.2, retention p < 0.05), reported as Odds Ratios (OR) with 95% CI.

Additionally, we conducted a survival analysis among patients with isolated *Candida* spp. in peritoneal fluid according to early antifungal therapy initiation, using weighted Cox proportional hazards model, with results reported as weighted Hazard Ratios (HR) with their corresponding 95% confidence intervals (CI). Survival probabilities were graphically represented through weighted Kaplan-Meier curves. As sensitivity analyses, alternative thresholds were assessed to define early antifungal therapy initiation and time-dependent weighted Cox model were conducted to assess potential time-dependent bias.

Missing data were imputed using a Monte Carlo-Markov-chained algorithm (MICE), assuming that data were randomly missing at baseline (five datasets generated, combined using Rubin’s rules). Only missing variables at baseline were used for the imputation process.

Collinearity between variables included in all multivariate models was checked using Variance Inflation Factor (VIF) and only non-collinear variables (VIF=<5) were included in the final models.

Proportional hazards assumption of weighted Cox regression models was verified using Schoenfeld residuals.

All statistical tests were two-tailed with a p-value<0.05 considered as significant. Analyses were performed on R statistical software, version 4.0.4 (https://cran.rproject.org/), and affiliated packages.

## Results

### Study population

During the study period, 383 patients were admitted to the participating ICUs for peritonitis. Of note, 4 patients with candidemia and 1 patient with *Candida spp.* isolated from both blood cultures and peritoneal fluid were excluded from the main analysis. Thus, 378 patients remained in the main analysis (Table 1 and **Supplementary Fig.** 1). Among them, 81 patients (21.4%) had *Candida* spp. isolated from peritoneal fluid cultures. No patient had a 1–3 B-D-glucan testing performed. No difference on immunosuppression status was observed between patients with isolated *Candida* spp. in peritoneal fluid or not (46.9% vs 46.8%, p = 1.000). Patients with isolated *Candida* spp. had a different acquisition setting (p = 0.045), presenting less often with both community-acquired (44.4% vs 51.5%) or early-onset nosocomial peritonitis (17.3% vs 23.9%), but more frequently with late-onset nosocomial peritonitis (38.3% vs 24.6%). Patients with isolated *Candida* spp. received more often antimicrobial therapy within the last 3 months before admission (30.9% vs 16.8%; p = 0.008) and presented more often with malnutrition (64.2% vs 49.8%; p = 0.030). Moreover, peritonitis from upper gastrointestinal tract origin was more frequent in *Candida*-positive patients compared to controls (44.4% vs 30.0%; p = 0.028).

**Table 1:** Baseline characteristics of patients with peritonitis according to *Candida* spp. isolation (raw and weighted populations).

	*Raw population*					*Weighted population*			
	All patients n = 378	No *Candida* spp. isolation n = 297	*Candida* spp. isolation n = 81	*p* value	*SMD*	No *Candida* spp. isolation n = 297.8	*Candida* spp. isolation n = 72.7	*p* value	SMD
**Baseline features**									
Age (years)	69 [60−75]	69 [61−75]	68 [59−75]	0.549	0.093	68 [61−75]	68 [59−75]	0.798	0.016
Female sex	131 (34.7)	101 (34.0)	32 (37.2)	0.674	0.030	102.1 (34.3)	24.1 (33.1)	0.861	0.011
**Comorbidities**									
Obesity (BMI > 30 kg/m^2^)	99 (26.2)	82 (27.6)	17 (21/0)	0.290	0.066	78.7 (26.4)	19.1 (26.5)	0.995	<0.001
Malnutrition	200 (52.9)	148 (49.8)	52 (64.2)	0.030	0.144	158.9 (53.3)	42.3 (58.2)	0.500	0.048
Cirrhosis	32 (8.5)	27 (9.1)	5 (6.2)	0.541	0.029	25.5 (8.6)	6.7 (9.3)	0.879	0.007
Diabetes	82 (21.7)	61 (20.5)	21 (25.9)	0.373	0.054	66.2 (22.2)	18.6 (25.6)	0.587	0.033
Chronic kidney failure	22 (5.8)	21 (7.1)	1 (1.2)	0.085	0.058	17.3 (5.8)	2.5 (3.5)	0.602	0.023
Congestive heart failure	41 (10.8)	30 (10.1)	11 (13.6)	0.490	0.034	32.7 (11.0)	6.7 (9.3)	0.626	0.017
Any immunosuppression	177 (46.8)	139 (46.8)	38 (46.9)	1.000	0.001	139.4 (46.8)	35.3 (48.5)	0.810	0.017
Solid organ transplantation	9 (2.4)	8 (2.7)	1 (1.2)	0.725	0.015	7.1 (2.4)	1.2 (1.6)	0.713	0.008
Hematological malignancy	21 (5.6)	18 (6.1)	3 (3.7)	0.584	0.024	16.4 (5.5)	2.9 (4.0)	0.623	0.015
Solid tumor	117 (31.0)	91 (30.6)	26 (32.1)	0.907	0.015	92.4 (31.0)	24.0 (33.0)	0.768	0.020
Long term corticosteroids	34 (9.0)	29 (9.8)	5 (6.2)	0.434	0.036	26.6 (8.9)	5.5 (7.6)	0.761	0.014
Antimicrobial therapy within the last 3 months	75 (19.8)	50 (16.8)	25 (30.9)	0.008	0.141	58.8 (19.7)	15.6 (21.5)	0.736	0.017
**Features of intra-abdominal infection**									
Setting of infection acquisition				0.045				0.877	
‘Community-acquired’ peritonitis	189 (50.0)	153 (51.5)	36 (44.4)		0.071	148.7 (49.9)	33.9 (46.7)		0.032
‘Early-onset nosocomial’ peritonitis (<7 days)	85 (22.5)	71 (23.9)	14 (17.3)		0.066	66.9 (22.4)	16.6 (22.8)		0.004
‘Late-onset nosocomial’ peritonitis (>7 days)	104 (27.5)	73 (24.6)	31 (38.3)		0.137	82.3 (27.6)	22.2 (30.6)		0.003
Postoperative peritonitis	139 (36.8)	103 (34.7)	36 (44.4)	0.137	0.098	109.2 (36.7)	28.7 (39.5)	0.678	0.028
Upper GI tract source^a^	127 (33.6)	89 (30.0)	36 (44.4)	0.028	0.139	102.1 (34.3)	26.5 (36.5)	0.733	0.022
Diffuse peritonitis^b^	243 (64.3)	184 (62.0)	59 (72.8)	0.093	0.109	191.3 (64.2)	49.2 (67.7)	0.615	0.034
**Initial severity and management**									
Time from hospital admission and peritonitis (days)	3 [0−9]	2 [0−7]	4 [0−12]	0.053	0.096	2 [0−9]	3 [0−9]	0.662	0.044
Sepsis at ICU admission	114 (30.2)	89 (30.0)	25 (30.9)	0.984	0.009	90.6 (30.4)	23.6 (32.5)	0.756	0.020
Septic shock at ICU admission	181 (47.9)	141 (47.5)	40 (49.4)	0.858	0.019	140.2 (47.1)	34.2 (47.1)	0.998	<0.001
SAPS II^c^	49 [37−61]	48 [37−61]	49 [37−59]	0.975	0.017	47 [37−61]	49 [37−63]	0.863	0.008
Timing of source control intervention^d^				0.701				0.977	
‘Emergency’ (<2H)	64 (16.9)	52 (17.5)	12 (14.8)		0.027	49.4 (16.6)	11.2 (15.5)		0.011
‘Urgent’ (2−6 H)	183 (48.4)	145 (48.8)	38 (46.9)		0.019	144.9 (48.7)	35.8 (49.3)		0.007
‘Delayed ‘(>6H)	131 (34.7)	100 (33.7)	31 (38.3)		0.046	103.5 (34.7)	25.6 (35.2)		0.005

Data are presented as median [Q1–Q3] and n (%).

Abbreviations: BMI: Body Mass Index, GI: Gastro-intestinal, ICU: Intensive Care Unit; SAPSII: Simplified Acute Physiology Score II, SMD: Standardized Mean Difference.

Missing values: ^a^ n = 24; ^b^ n = 33; ^c^ n = 24; ^d^ n = 36.

### Outcomes according to *Candida* spp. isolation in peritoneal fluid

Survival up to day-90 along with clinical courses in the raw population did not differ between groups (Log-rank test; p = 0.49) (**Supplementary Fig.** 2 **and** Table 2).

**Table 2:** Clinical course of patients with peritonitis according to *Candida* spp. isolation (raw and weighted populations).

	*Raw population*			*Weighted population*			
	All patients n = 378	No *Candida* spp. isolation n = 297	*Candida* spp. isolation n = 81	*p* value	No *Candida* spp. isolation n = 297.8	*Candida* spp. isolation n = 72.7	*p* value
**Source control achievement**							
Successful source control at day 7*^a^*	210 (55.7)	170 (57.4)	40 (49.4)	0.244	168.9 (56.9)	37.4 (51.5)	0.441
Source control failure at day 7, persistent inflammation*^b^*	165 (51.6)	129 (50.6)	36 (55.4)	0.581	129.3 (50.7)	32.6 (55.3)	0.544
Source control failure at day 7, additional radiological intervention*^c^*	19 (5.6)	12 (4.5)	7 (10.0)	0.135	13.3 (4.9)	6.5 (10.3)	0.127
Source control failure at day 7, additional surgical intervention*^d^*	87 (25.7)	68 (25.3)	19 (27.1)	0.869	69.9 (26.0)	19.4 (30.6)	0.504
Additional intervention required within 28 days after peritonitis onset	114 (30.2)	90 (30.3)	24 (29.6)	1.000	91.2 (30.6)	23.6 (32.5)	0.775
**Organ dysfunctions and life-sustaining therapies**							
Invasive mechanical ventilation	275 (72.8)	212 (71.4)	63 (77.8)	0.315	213.4 (71.6)	56.9 (78.3)	0.263
Invasive mechanical ventilation duration*^e^*	3 [1−8]	3 [1−8]	3 [1−8]	0.962	3 [1−9]	3 [1−8]	0.684
Acute Respiratory Distress Syndrome	69 (18.3)	59 (19.9)	10 (12.3)	0.164	59.7 (20.0)	7.9 (10.8)	0.100
Vasopressors use	311 (82.3)	242 (81.5)	69 (85.2)	0.542	242.5 (81.4)	62.2 (85.5)	0.422
Vasopressors duration *^f^*	2 [1−4]	2 [1−4]	2 [1−5]	0.569	2 [1−4]	2 [1−4]	0.637
Acute kidney injury	263 (69.6)	208 (70.0)	55 (67.9)	0.815	209.1 (70.2)	49.4 (67.9)	0.717
AKI KDIGO stage *^g^*				0.436			0.922
*Stage I*	52 (19.5)	38 (17.9)	14 (25.5)		39.4 (18.5)	10.2 (20.7)	
*Stage II*	59 (22.1)	47 (22.2)	12 (21.8)		47.2 (22.1)	11.3 (22.9)	
*Stage III*	156 (58.4)	127 (59.9)	29 (52.7)		126.7 (59.4)	27.9 (56.4)	
Renal Replacement Therapy	83 (22.0)	64 (21.5)	19 (23.5)	0.829	62.6 (21.0)	16.6 (22.8)	0.763
Antibiotics duration*^h^*	7 [5−10]	7 [5−9]	7 [5−10]	0.451	7 [5−9]	7 [5−10]	0.631
**Outcomes**							
ICU length of stay	7 [3−17]	7 [3−17]	8 [3−16]	0.823	7 [3−17]	8 [3−16]	0.853
Hospital length of stay*^i^*	26 [14−49]	25 [13−48]	27 [16−50]	0.749	25 [13−48]	27 [15−43]	0.945
ICU mortality	106 (28.0)	78 (26.3)	28 (34.6)	0.182	78.6 (26.4)	23.5 (32.4)	0.344
In-hospital mortality	125 (33.1)	94 (31.6)	31 (38.3)	0.322	95.4 (32.0)	25.2 (34.7)	0.684

Data are presented as median [Q1–Q3] and n (%).

Abbreviations: AKI: Acute Kidney Injury, KDIGO: Kidney Disease Improving Global Outcomes, ICU: Intensive Care Unit.

Missing values: ^a^ n = 1; ^b^ n = 58, ^c^ n = 40, ^d^ n = 39, ^e^ n = 75, ^f^ n = 57, ^g^ n = 111, ^h^ n = 9, ^i^ n = 19.

**Fig. 1:**
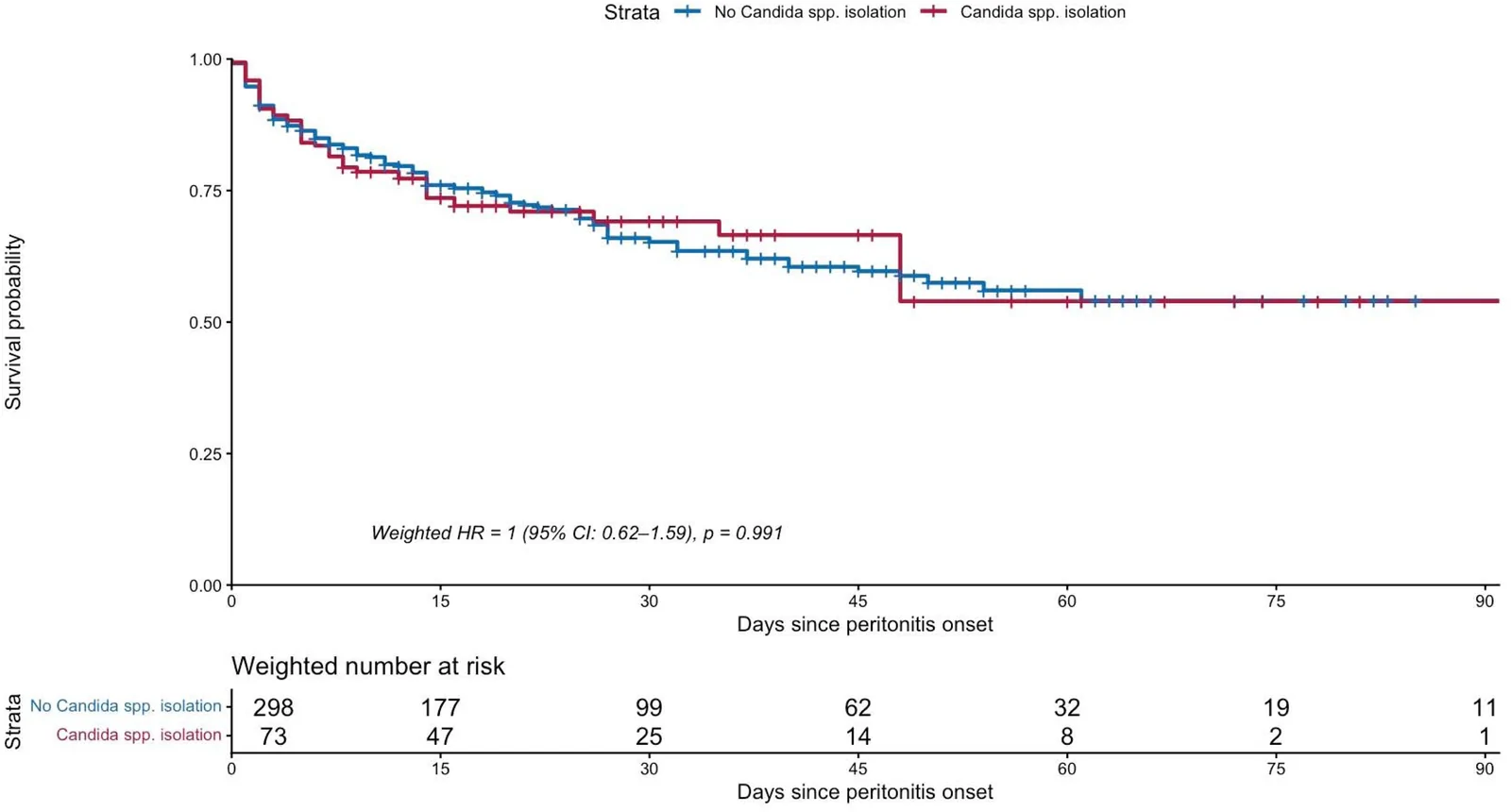
Weighted survival analysis of critically ill patients with peritonitis according to *Candida* spp. isolation.

To address potential confounding due to baseline imbalances, we implemented a stabilized IPTW approach. After weighting, all covariates had adequate balance (SMD < 0.2) and PS density had better overlap (63.8% [53.3%–73.2%] vs 81% [67.8%–92.1%], p < 0.001) (Table 1, **Supplementary Table** 2**, Supplementary Figs.** 3 **and** 4). Propensity score model showed some optimism on bootstrap validation and a low event-per-variable ratio, consistent with the relatively large number of baseline covariates included. In the weighted Cox proportional hazards model, 90-day all-cause mortality did not differ according to *Candida* spp. isolation in peritoneal fluid (weighted HR 1.00, 95% CI 0.62–1.59; p = 0.991) (Fig. 1). All secondary outcomes were similar between groups (Table 2).

All sensitivity analyses including the five patients with concomitant candidemia provided similar results regarding mortality and all secondary outcomes (**Supplementary Table** 3,4 **& Supplementary Fig.** 5).

### Risk factors for *Candida* spp. isolation in peritoneal fluid

In backward multivariate analysis antimicrobial therapy within the last 3 months (adjusted OR 2.15, 95% CI 1.20–3.81; p = 0.009), malnutrition (adjusted OR 1.84, 95% CI 1.10–3.14; p = 0.022), upper gastrointestinal origin (adjusted OR 2.04, 95% CI 1.20–3.44; p = 0.008) and diffuse peritonitis (adjusted OR 1.75, 95% CI 1.01–3.13; p = 0.049) were independently associated with *Candida* spp. isolation in peritoneal fluid (**Supplementary Table** 5). Sensitivity analysis including patients with concomitant candidemia provided similar results (**Supplementary Table** 6).

### Survival analysis in patients with isolated *Candida* spp. and effect of early antifungal therapy

Among the 81 patients with isolated *Candida* spp. in peritoneal fluid, 31 patients (38.3%) received early antifungal therapy (Table 3). Of note, 50 patients (61.7%) did not receive early antifungal therapy, including 18 patients (21.0%) who never received antifungal therapy. These never-treated patients were less frequently women (p = 0.029) and presented less frequently with upper GI tract origin of peritonitis (p = 0.030) compared to early-treated and late-treated patients. Median time to antifungal initiation was 0 [0-1] days in early antifungal therapy initiation group compared to 3 [3-5] days in no early antifungal therapy initiation group (p < 0.001). In early-treated group, antifungal agents prescribed upfront differed across institutions (p = 0.025), with varying use of echinocandins (40 to 100%), whereas antifungal agents used for de-escalation were similar across centers (p = 1.000), mainly with Fluconazole (75 to 100%). However, all *Candida* spp. strains were sensitive to the early-initiated antifungal therapy. Thus, no patient was classified in the “No early antifungal therapy” group due to resistance to upfront antifungal therapy.

**Table 3:** Baseline characteristics of patients with *Candida* spp. isolation according to early antifungal therapy (raw and weighted population).

	*Raw population*					*Weighted population*			
	All patients n = 81	No early antifungal therapy n = 50	Early antifungal therapy n = 31	*p* value	*SMD*	No early antifungal therapy n = 42.12	Early antifungal therapy n = 22.09	*p* value	SMD
**Baseline features**									
Age (years)	68 [59−75]	69 [59−75]	66 [58−73]	0.363	0.262	68 [57−75]	70 [56−73]	0.820	0.125
Female sex	30 (37.0)	15 (30.0)	15 (48.4)	0.153	0.184	15.6 (36.9)	11.0 (49.6)	0.345	0.127
**Comorbidities**									
Obesity (BMI > 30)	17 (21.0)	8 (16.0)	9 (29.0)	0.263	0.130	7.1 (16.9)	4.5 (20.4)	0.709	0.035
Malnutrition	52 (64.2)	31 (62.0)	21 (67.7)	0.775	0.057	25.1 (59.7)	14.2 (64.3)	0.723	0.046
Diabetes	21 (25.9)	9 (18.0)	12 (38.7)	0.071	0.207	8.9 (21.0)	6.9 (31.4)	0.362	0.103
Any immunosuppression	38 (46.9)	23 (46.0)	15 (48.4)	1.000	0.024	18.0 (42.6)	10.0 (45.1)	0.848	0.025
Solid tumor	26 (32.1)	18 (36.0)	8 (25.8)	0.478	0.102	13.9 (33.0)	6.4 (28.8)	0.737	0.042
Antimicrobial therapy within the last 3 months	25 (30.9)	16 (32.0)	9 (29.0)	0.973	0.030	14.1 (33.5)	7.1 (32.2)	0.918	0.013
**Features of intra-abdominal infection**									
Setting of infection acquisition				0.015				0.475	
‘Community-acquired’ peritonitis	36 (44.4)	16 (32.0)	20 (64.5)		0.325	16.7 (39.8)	12.4 (56.0)		0.162
‘Early-onset nosocomial’ peritonitis (<7 days)	14 (17.3)	10 (20.0)	4 (12.9)		0.071	7.2 (17.1)	2.8 (12.7)		0.044
‘Late-onset nosocomial’ peritonitis (>7 days)	31 (38.3)	24 (48.0)	10 (32.3)		0.254	18.2 (43.1)	6.9 (31.3)		0.118
Postoperative peritonitis	36 (44.4)	26 (52.0)	10 (32.3)	0.132	0.197	19.8 (46.9)	7.1 (32.0)	0.239	0.150
Source of peritonitis: Upper GI tract^a^	36 (44.4)	17 (34.0)	19 (61.3)	0.030	0.272	15.2 (36.3)	10.8 (48.8)	0.342	0.125
Diffuse peritonitis^b^	59 (72.8)	345(70.0)	24 (77.4)	0.636	0.074	30.3 (71.9)	16.9 (76.6)	0.701	0.046
**Initial severity and management**									
Time from hospital admission and peritonitis (days)	4 [0−12]	7 [1−13]	0 [0−7]	0.019	0.113	4 [0−13]	0 [0−9]	0.236	0.093
Sepsis at ICU admission	25 (30.9)	18 (36.0)	7 (22.6)	0.306	0.134	15.0 (35.5)	6.5 (29.6)	0.701	0.085
Septic shock at ICU admission	40 (49.4)	20 (40.0)	20 (64.5)	0.055	0.245	17.9 (42.4)	11.7 (53.0)	0.431	0.059
SAPS II^c^	49 [37−59]	51 [36−59]	49 [40−61]	0.823	0.056	49 [33−59]	49 [39−58]	0.662	0.100
Timing of source control intervention^d^				0.203				0.760	
‘Emergency’ (<2H)’	12 (14.8)	5 (10.0)	7 (22.6)		0.126	6.1 (14.5)	4.8 (21.6)		0.071
‘Urgent’ (2−6 H)	38 (46.9)	23 (46.0)	15 (48.4)		0.024	18.0 (42.6)	9.3 (42.1)		0.005
‘Delayed’ (>6H)	31 (38.3)	20 (40.0)	9 (29.0)		0.150	18.1 (42.9)	8.0 (36.3)		0.066
**Microbiology findings**									
Yeast-positive direct examination of peritoneal fluid	16 (19.8)	10 (20.0)	6 (19.4)	1.000	0.006	9.1 (21.5)	5.1 (23.2)	0.880	0.017
Pure *Candida* spp. culture	17 (21.0)	8 (16.0)	9 (29.0)	0.263	0.130	6.0 (14.2)	4.1 (18.6)	0.577	0.044
*Candida albicans*	62 (76.5)	40 (80.0)	22 (71.0)	0.508	0.090	32.9 (78.1)	16.8 (76.1)	0.849	0.020
Fluconazole-resistant isolate	7 (8.6)	5 (11.4)	2 (7.1)	0.856	0.035	5.6 (13.3)	2.7 (12.2)	0.910	0.011

Data are presented as median [Q1–Q3] and n (%).

Abbreviations: BMI: Body Mass Index, GI: Gastro-intestinal, ICU: Intensive Care Unit; SAPSII: Simplified Acute Physiology Score II.

Missing values: ^a^ n = 3; ^b^ n = 4; ^c^ n = 6; ^d^ n = 6.

Before IPTW, patients receiving early antifungal therapy presented more often with upper gastrointestinal tract origin of peritonitis (61.3% vs 34.0%, p = 0.030). Acquisition setting differed between groups (p = 0.015), as patients receiving early antifungal therapy presented more often with community-acquired peritonitis (64.5% vs 32.0%) and less frequently with early-onset (12.9% vs 20.0%) or late-onset nosocomial peritonitis (22.6% vs 48.0%). All other baseline characteristics were similar between groups. *Candida albicans* accounted for most isolates (76.5%), followed by *Candida glabrata* (11.5%). Overall, 11.5% of patients had a fluconazole-resistant strain, while no strain was resistant to echinocandins.

Survival up to day-90 after peritonitis onset was not associated with early antifungal therapy (log-rank test, p = 0.16) (**Supplementary Fig.** 6).

Source control achievement, organ dysfunctions and the use of life-sustaining therapies did not differ whether patients with isolated *Candida* spp. in peritoneal fluid received early antifungal therapy or not (**Supplementary Table** 7).

**Fig. 2:**
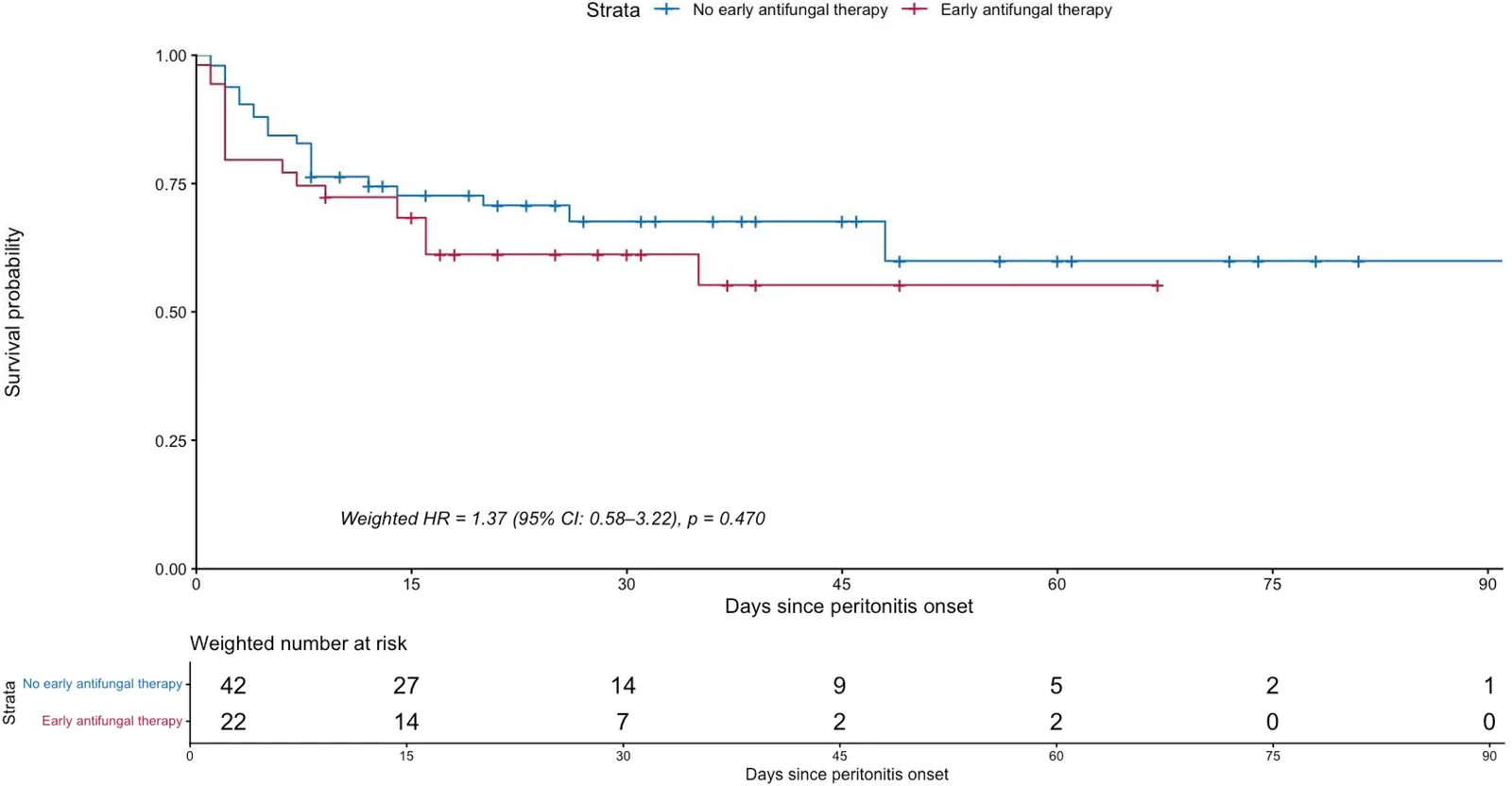
Weighted survival analysis of critically ill patients with isolated *Candida* spp. according to early antifungal therapy.

After IPTW, baseline characteristics included in the weighting process had adequate balance (SMD < 0.2) (Table 3**, Supplementary Fig.** 7). and PS density had better overlap (65.7% [37.6%–87.6%] vs 38.9% [21.2%–54.9%], p = 0.004) (**Supplementary Fig.** 8**, Supplementary Table** 2). Early antifungal therapy was not associated with 90-day all-cause mortality (weighted HR 1.37, 95% CI 0.58–3.22; p = 0.470) (Fig. 2). Similarly, clinical courses did not differ between groups, including antifungals duration (**Supplementary Table** 7).

### Sensitivity analyses

First, among patients with no *Candida* spp. isolated in peritoneal fluid, 32 patients received early antifungal therapy despite subsequently negative cultures. Compared to patients with no *Candida* spp. isolated and no early antifungal therapy, these patients presented more often with septic shock (p = 0.047) but did not differ on other baseline characteristics. Survival up to day-90 was not associated with early antifungal therapy (Log rank, p = 0.99)

Second, among patients with isolated *Candida* spp., sensitivity analyses including patients with concomitant candidemia provided consistent results regarding mortality and clinical courses according to early antifungal therapy (**Supplementary Table** 8,9**, Supplementary Fig.** 9).

Moreover, survival up to day-90 after peritonitis onset did not differ when early antifungal initiation was defined using alternative thresholds of 24 h, 72 h, and 6 days, respectively (**Supplementary Figs.** 10–12).

Furthermore, early antifungal therapy initiation was not associated with mortality when categorizing patients as “early-treated” (< = 48 h), “late-treated” (>48 h) or “never-treated” (Log rank test, p = 0.083) (**Supplementary Fig.** 13).

Last, when considering early antifungal therapy initiation as a time-dependent variable, we found no association with 90-day all-cause mortality in a time-dependent weighted Cox model (weighted HR for early antifungal therapy initiation, 1.81 (95% CI 0.31–10.43), p = 0.509; weighted HR for tt(early antifungal therapy initiation), 0.82 (95% CI 0.36–1.88), p = 0.636).

### Subgroup analyses

Early antifungal therapy was not associated with 90-day all-cause mortality, neither in patients with nosocomial peritonitis (Log rank, p = 0.45), nor in most severe patients presenting with septic shock (Log rank, p = 0.82) (**Supplementary Figs.** 14,15). In the subgroup of patients with yeast-positive direct examination or pure *Candida* spp. cultures, we observed a numerically lower rate of death in patients receiving early-antifungal treatment (60.0% vs 83.3%) without statistical significance (p-value = 0.676).

## Discussion

In this multicenter cohort of critically ill patients with secondary peritonitis, *Candida* spp. isolation in peritoneal fluid was frequent but was not independently associated with 90-day all-cause mortality after adjustment using inverse probability of treatment weighting. Moreover, among patients with isolated *Candida* spp. in peritoneal fluid, early antifungal therapy was not associated with improved 90-day survival.

The prevalence of *Candida* spp. in our cohort is significantly higher than previously reported, affecting nearly a quarter of the patients studied [4]. Systematic intraoperative peritoneal sampling may explain the higher rate of *Candida* spp. isolation compared with studies relying on clinically driven cultures.

In the setting of peritonitis, Dupont et al. reported a dedicated bedside scoring system to predict *Candida* spp. isolation, including four risk factors (female sex, septic shock, upper-gastrointestinal origin and/or previous exposure to antibiotics for more than 48 h) [35], that have been endorsed in French national guidelines [12]. While we identified several risk factors for *Candida* spp. isolation (namely malnutrition, antimicrobial therapy within the last 3 month, upper GI tract source and diffuse peritonitis), these findings should not be interpreted as an argument for systematic empiric antifungal therapy. Our results suggest that early antifungal treatment is not associated 90-day mortality in patients with peritoneal *Candida* spp. isolation without candidemia, regardless of the presence of risk factors. Thus, rather than guiding treatment decisions, these risk factors may help inform diagnostic strategies (e.g., systematic intraoperative sampling) and identify patients who warrant closer monitoring.

While the life-threatening condition of candidemia is well established in the literature, the potential role of *Candida* spp. in peritonitis is less depicted. By analogy to candidemia, historical management strategies and major guidelines on severe peritonitis have generally favored an interventionist approach to *Candida* spp., arguing for documented treatment when *Candida* spp. in peritoneal fluid, and even supporting empirical antifungal treatment based on patient severity [13] or in selected high-risk situations [12,14]. This dogma has been challenged recently by the FUNDICU consortium [21]. In these definitions, positive specimen obtained from peritoneal fluid was not sufficient to be considered as mycological criteria to diagnose probable deep-seated candidiasis. This paradigm shift suggests that, in the setting of peritonitis with rapid source control and without concomitant *Candida* spp. bloodstream infection, isolated *Candida* spp. in peritoneal fluid could stand as a transient contaminant rather than a true pathogen. In our large multicenter study, we did not show any difference in 90-day all-cause mortality among patients with isolated *Candida* spp. in peritoneal fluid or not. Furthermore, we found no difference in 90-day all-cause mortality among patients with isolated *Candida* spp. in peritoneal fluid, whether they received early antifungal therapy or not. Empiric antifungal strategies in intra-abdominal infections have largely been extrapolated from candidemia, where delayed therapy is clearly associated with increased mortality [[16], [17], [18], [19], [20]]. Whether this association is transposable for patients with peritonitis with isolated *Candida* spp. in peritoneal fluid without concomitant candidemia is uncertain. Several previous studies have addressed this question with conflicting results [7,10,11,[24], [25], [26], [27]]. Our study provides contemporary data (2020–2022) reflecting current practices, and specifically addresses the unresolved dilemma of peritoneal *Candida* spp. isolation without candidemia. Using IPTW, we found no association with mortality and no benefit of early antifungal therapy, aligning with the recent FUNDICU consensus definitions. These findings support a more restrictive approach, while highlighting the need for further prospective studies. The ongoing CASPER multicenter randomized trial would probably give valuable answers in this regard (NCT03580733). Therefore, these data suggest that routine early antifungal therapy based solely on peritoneal *Candida* spp. isolation may lead to overtreatment without clear clinical benefit. Of note, the rate of positive direct examination of peritoneal fluid was similar between patients who received early antifungal therapy and those who did not. From a clinical perspective, our results support a more individualized approach to antifungal therapy in secondary peritonitis, rather than systematic empiric treatment. In our study, we found no association between 90-day all-cause mortality and early antifungal therapy in the subgroup of patients with nosocomial peritonitis, nor in the subgroup of the most severe patients presenting with septic shock. However, these subgroups were limited by small sample size and should therefore be interpreted cautiously as exploratory analyses. Yeast-positive direct examination has been associated with fungal burden and higher mortality in previous literature [6]. Unfortunately, due to very limited sample size, we cannot properly assess potential benefit of early antifungal therapy initiation in patients with a yeast-positive direct examination, or pure *Candida* spp. cultures in our study although we observed a numerically higher survival rate in patients receiving early antifungal treatment. Thus, further work should focus on identifying which patients could benefit from antifungal therapy when *Candida* spp. is isolated only in peritoneal fluid.

While our study provides clinically relevant insights into the management of peritonitis with isolated *Candida* spp., several limitations should be acknowledged to contextualize these findings. First, its retrospective and observational design precludes definitive causal inferences, despite our robust methodological approach to control for confounding. Second, despite multicenter inclusion over a three-year period, the sample size remains limited, in line with prior studies. This limitation reduces statistical power and the precision of most estimates, as illustrated by wide confidence intervals that do not fully exclude a potential benefit or harm. Nevertheless, this study is among the largest specifically addressing *Candida* spp. isolation in the setting of secondary peritonitis. Third, we cannot fully exclude any misclassification bias related to heterogeneous fungal work-up among participant centers, particularly in community-acquired peritonitis, where low-burden co-isolates may not be systematically assessed and reported. Such nondifferential misclassification would tend to bias associations toward the null and reduce statistical power. Moreover, we did not collect a standardized assessment of the quality of the initial source control, that could represent a source of residual confounding. Fourth, only 4 patients with isolated *Candida* spp. were treated with surgery more than 24 h after peritonitis onset, so that culture positivity would be sufficient to define invasive candidiasis according to FUNDICU criteria. Due to very few cases, we cannot restrict the main analysis or perform subgroup analysis on these patients. Thus, our results apply to patients with early source control with subsequent *Candida* spp. isolation in surgical specimens, and not to patients with deep-seated candidiasis according to FUNDICU criteria. Fifth, decision to administer early antifungal therapy was left at discretion of the treating physician, who may have been more likely to prescribe antifungal treatment in the most severely ill patients (e.g. septic shock). Although our weighting strategy achieved acceptable balance between groups with respect to objective baseline characteristics at ICU admission, it could not account for changes in clinical status between ICU admission and treatment initiation. Therefore, we could not fully exclude any residual indication bias on decision to start early antifungal therapy or not. This may partly explain the higher, although not statistically significant, 90-day mortality in early treated patients with isolated *Candida* spp. Last, our weighting strategy included a relatively large number of baseline variables to achieve the best covariate balance between groups. Given the limited number of events, this approach may have increased the risk of model instability and overfitting. We therefore explored more parsimonious weighting models, but these did not achieve adequate post-weighting balance for several key covariates. Accordingly, we favored the specification that provided the best covariate balance, while acknowledging the potential trade-off with model parsimony.

In conclusion, among critically ill patients with peritonitis, *Candida* spp. isolation in peritoneal fluid is not associated with 90-day all-cause mortality. Among patients with isolated *Candida* spp. in peritoneal fluid, early antifungal therapy is not associated with 90-day all-cause mortality. These findings should be interpreted with caution given the retrospective design and the limited sample size, which is reflected by wide confidence intervals that do not fully exclude a clinically meaningful benefit or harm. Nonetheless, our results suggest that systematic empiric antifungal therapy in this setting may warrant reappraisal, and future prospective studies are needed to identify patients who truly benefit from antifungal treatment.

## Contributors

M.M., C.A., M.B., Y.L. and F.R. conceived, designed and supervised the study. C.A., M.B., P.F., P.B., G.R., Y.F., JP.G, R.P., A.D., Y.L. and F.R collected the data and revised the manuscript. M.M. & FR performed the statistical analysis. M.M. & FR wrote the first draft of the article. All authors interpreted the data, revised the manuscript and approved the final version of the manuscript before submission.

## Ethical considerations

The study was carried out in accordance with the ethical standards of the Declaration of Helsinki and was approved by the “Ethics Committee for Research in Anesthesia and Intensive Care” (CESAR) of the “French Society of Anesthesia and Intensive Care” (SFAR) on February 21^th^, 2023 (Registration number: IRB 00010254 - 2023 - 017).

## Data availability

The datasets from this study are available from the corresponding author on request.

## Declaration of competing interest

The authors report no conflict of interest related to this work.

J-PG received honoraria from Gilead, MundiPharma, Pfizer, and Shionogi for talks and conferences in congresses.

## Footnotes

Appendix A Supplementary material related to this article can be found, in the online version, at doi:https://doi.org/10.1016/j.aicoj.2026.100140.
