# Supplementary material for: Prognostic impact of Candida spp. isolation and early antifungal therapy in ICU patients with peritonitis: A multicenter cohort study
Source: Ann Intensive Care. 2026 Aug 25;16:100140. doi: 10.1016/j.aicoj.2026.100140 (PMC13572066; doi:10.1016/j.aicoj.2026.100140)
Supplement: Supplementary file 1 [file mmc1.docx]

**Supplementary Table 1.** ICD-10 codes used for screening of patients with peritonitis.

| **ICD-10 code** | **Description** |
| --- | --- |
| K65 | Peritonitis |
| N73.5 | Female pelvitic peritonitis, unspecified |
| K35.2 | Acute appendicitis with generalized peritonitis |
| K35.3 | Acute appendicitis with localized peritonitis |
| K66.8 | Other unspecified disorders of the peritoneum |

**Supplementary Table 2. Patient baseline characteristics included in propensity-score (PS) models**

| **Model 1^a^** | **Model 2^b^** |
| --- | --- |
| Age (years) | Age (years) |
| Female sex | Female sex |
| Obesity (BMI>30) | Obesity (BMI>30) |
| Malnutrition | Malnutrition |
| Cirrhosis | - |
| Diabetes | Diabetes |
| Chronic kidney failure | - |
| Congestive heart failure | - |
| Any immunosuppression | Any immunosuppression |
| Solid organ transplantation | - |
| Hematological malignancy | - |
| Solid tumor | Solid tumor |
| Long term corticosteroids | - |
| Antimicrobial therapy within the last 3 months | Antimicrobial therapy within the last 3 months |
| Setting of infection acquisition | Setting of infection acquisition |
| Postoperative peritonitis | Postoperative peritonitis |
| Source of peritonitis: Upper GI tract | Source of peritonitis: Upper GI tract |
| Diffuse peritonitis | Diffuse peritonitis |
| Time from hospital admission and peritonitis (days) | Time from hospital admission and peritonitis (days) |
| Sepsis at ICU admission | Sepsis at ICU admission |
| Septic shock at ICU admission | Septic shock at ICU admission |
| SAPS II | SAPS II |
| Timing of source control intervention | Timing of source control intervention |
| - | Yeast-positive direct examination of peritoneal fluid |
| - | Pure *Candida* spp. culture |
| - | *Candida albicans* |
| - | Fluconazole-resistant isolate |

All patient characteristics at baseline with at least 5 patients per group were considered for inclusion in non‑parsimonious multivariable logistic regression models to estimate propensity scores

^a^ Propensity score Model 1 estimated the probability of *Candida* spp. isolation.

^b^ Propensity score Model 2 estimated the probability of early antifungal therapy.

**Supplementary Table 3.** Baseline characteristics of patients with peritonitis according to *Candida* spp. isolation, including patients with candidemia (raw and weighted populations)

|  | *Raw population* | | | | | *Weighted population* | | | |
| --- | --- | --- | --- | --- | --- | --- | --- | --- | --- |
|  | **All patients**  **n=383** | **No *Candida* spp*.* isolation**  **n=297** | ***Candida* spp*.***  **isolation**  **n=86** | ***p* value** | ***SMD*** | **No *Candida* spp*.* isolation**  **n=294.3** | ***Candida* spp*.***  **isolation n=73.6** | ***p* value** | **SMD** |
| **Baseline features** |  |  |  |  |  |  |  |  |  |
| Age (years) | 69 [60-75] | 69 [61-75] | 68 [58-74] | 0.462 | 0.102 | 68 [61-75] | 68 [59-74] | 0.662 | 0.044 |
| Female sex | 133 (34.7) | 101 (34.0) | 32 (37.2) | 0.674 | 0.032 | 102.0 (34.7) | 25.4 (34.5) | 0.983 | 0.001 |
| **Comorbidities** |  |  |  |  |  |  |  |  |  |
| Obesity (BMI>30kg/m^2^) | 99 (25.8) | 82 (27.6) | 17 (19.8) | 0.186 | 0.078 | 77.5 (26.3) | 17.5 (23.7) | 0.655 | 0.026 |
| Malnutrition | 203 (53.0) | 148 (49.8) | 55 (64.0) | 0.029 | 0.141 | 155.7 (52.9) | 44.7 (60.7) | 0.235 | 0.077 |
| Cirrhosis | 32 (8.4) | 27 (9.1) | 5 (5.8) | 0.456 | 0.032 | 25.0 (8.5) | 5.5 (7.5) | 0.792 | 0.011 |
| Diabetes | 83 (21.7) | 61 (20.5) | 22 (25.6) | 0.395 | 0.050 | 63.6 (21.6) | 17.9 (24.3) | 0.623 | 0.026 |
| Chronic kidney failure | 22 (5.7) | 21 (7.1) | 1 (1.2) | 0.070 | 0.059 | 17.1 (5.8) | 1.5 (2.0) | 0.271 | 0.037 |
| Congestive heart failure | 41 (10.7) | 30 (10.1) | 11 (12.8) | 0.608 | 0.027 | 31.5 (10.7) | 7.2 (9.7) | 0.782 | 0.010 |
| Any immunosuppression | 181 (47.3) | 139 (46.8) | 42 (48.8) | 0.833 | 0.020 | 138.6 (47.1) | 35.1 (47.7) | 0.924 | 0.006 |
| Solid organ transplantation | 9 (2.3) | 8 (2.7) | 1 (1.2) | 0.674 | 0.015 | 7.0 (2.4) | 1.3 (1.7) | 0.748 | 0.006 |
| Hematological malignancy | 22 (5.7) | 18 (6.1) | 4 (4.7) | 0.817 | 0.014 | 16.8 (5.7) | 3.2 (4.3) | 0.607 | 0.014 |
| Solid tumor | 119 (31.1) | 91 (30.6) | 28 (32.6) | 0.837 | 0.019 | 91.3 (31.0) | 24. (32.6) | 0.795 | 0.016 |
| Long term corticosteroids | 35 (9.1) | 29 (9.8) | 6 (7.0) | 0.564 | 0.028 | 26.8 (9.1) | 5.1 (6.9) | 0.554 | 0.022 |
| Antimicrobial therapy within the last 3 months | 77 (20.1) | 50 (16.8) | 27 (31.4) | 0.005 | 0.145 | 57.4 (19.5) | 17.9 (24.3) | 0.340 | 0.048 |
| **Features of intra-abdominal infection** | |  |  |  |  |  |  |  |  |
| Setting of infection acquisition |  |  |  | 0.024 |  |  |  | 0.689 |  |
| ‘Community-acquired’ peritonitis | 189 (49.3) | 153 (51.5) | 36 (41.9) |  | 0.097 | 145.9 (49.6) | 33.9 (46.0) |  | 0.036 |
| ‘Early-onset nosocomial’  peritonitis (<7 days) | 87 (22.7) | 71 (23.9) | 16 (18.6) |  | 0.053 | 67.5 (23.0) | 15.8 (21.5) |  | 0.015 |
| ‘Late-onset nosocomial’  peritonitis (>7 days) | 107 (27.9) | 73 (24.6) | 34 (39.5) |  | 0.150 | 80.8 (27.5) | 23.9 (32.5) |  | 0.051 |
| Postoperative peritonitis | 143 (37.3) | 103 (34.7) | 40 (46.5) | 0.061 | 0.118 | 108.5 (36.9) | 30.3 (41.1) | 0.499 | 0.042 |
| Upper GI tract source^a^ | 121 (33.6) | 89 (30.0) | 38 (44.2) | 0.019 | 0.147 | 100.0 (34.0) | 29.7 (40.3) | 0.304 | 0.063 |
| Diffuse peritonitis^b^ | 225 (64.1) | 185 (62.3) | 60 (69.8) | 0.252 | 0.108 | 185.8 (63.1) | 50.5 (68.7) | 0.382 | 0.055 |
| **Initial severity and management** | | |  |  |  |  |  |  |  |
| Time from hospital admission and peritonitis (days) | 3 [0-9] | 2 [0-7] | 4 [0-13] | 0.018 | 0.108 | 3 [0-9] | 3 [0-10] | 0.435 | 0.055 |
| Sepsis at ICU admission | 117 (30.5) | 89 (30.0) | 28 (32.6) | 0.744 | 0.026 | 90.5 (30.7) | 22.9 (31.1) | 0.956 | 0.003 |
| Septic shock at ICU admission | 183 (47.8) | 141 (47.5) | 42 (48.8) | 0.920 | 0.014 | 138.9 (47.2) | 35.7 (48.5) | 0.842 | 0.013 |
| SAPS II^c^ | 49 [38-61] | 48 [37-62] | 49 [39-59] | 0.912 | 0.053 | 48 [38-61] | 49 [39-63] | 0.700 | 0.033 |
| Timing of source control intervention^d^ | |  |  | 0.359 |  |  |  | 0.962 |  |
| ‘Emergency’ (<2H) | 52 (15.0) | 51 (17.2) | 10 (11.6) |  | 0.025 | 46.4 (15.8) | 10.6 (14.5) |  | 0.013 |
| ‘Urgent’ (2-6H) | 123 (49.7) | 144 (48.5) | 41 (47.7) |  | 0.009 | 146.3 (49.7) | 37.3 (50.6) |  | 0.008 |
| ‘Delayed ‘ (>6H) | 122 (35.3) | 102 (34.3) | 35 (40.7) |  | 0.035 | 101.5 (34.5) | 25.7 (34.9) |  | 0.004 |

Data are presented as median [Q1-Q3] and n (%).

Abbreviations: BMI: Body Mass Index, GI: Gastro-intestinal, ICU: Intensive Care Unit; SAPSII: Simplified Acute Physiology Score II, SMD: Standardized Mean Difference

Missing values: ^a^ n=32; ^b^ n=41; ^c^ n=26; ^d^ n=45

**Supplementary Table 4.** Clinical course of patients with peritonitis according to *Candida* spp. isolation, including patients with candidemia (raw and weighted populations)

|  | *Raw population* | | | *Weighted population* | | | |
| --- | --- | --- | --- | --- | --- | --- | --- |
|  | **All patients**  **n=383** | **No *Candida* spp*.* isolation**  **n=297** | ***Candida* spp*.***  **isolation**  **n=86** | ***p* value** | **No *Candida* spp*.* isolation**  **n=294.3** | ***Candida* spp*.***  **isolation n=73.6** | ***p* value** |
| **Source control achievement** |  |  |  |  |  |  |  |
| Successful source control at day 7*^a^* | 211 (55.2) | 170 (57.4) | 41 (47.7) | 0.139 | 167.0 (57.0) | 37.3 (50.6) | 0.325 |
| Source control failure at day 7, persistent inflammation*^b^* | 170 (52.3) | 129 (50.6) | 41 (58.6) | 0.294 | 128.0 (50.7) | 34.6 (57.1) | 0.371 |
| Source control failure at day 7, additional radiological intervention*^c^* | 19 (5.5) | 12 (4.5) | 7 (9.3) | 0.180 | 12.5 (4.7) | 6.8 (10.5) | 0.080 |
| Source control failure at day 7, additional surgical intervention*^d^* | 90 (26.2) | 68 (25.3) | 22 (29.3) | 0.577 | 69.0 (25.9) | 19.2 (29.9) | 0.522 |
| Additional intervention required within 28 days after peritonitis onset | 117 (30.5) | 90 (30.3) | 27 (31.4) | 0.952 | 90.2 (30.7) | 23.7 (32.2) | 0.793 |
| **Organ dysfunctions and life-sustaining therapies** |  |  |  |  |  |  |  |
| Invasive mechanical ventilation | 280 (73.1) | 212 (71.4) | 68 (79.1) | 0.201 | 211.2 (71.8) | 57.5 (78.1) | 0.267 |
| Invasive mechanical ventilation duration*^e^* | 3 [1-8] | 3 [1-8] | 3 [1-9] | 0.756 | 3 [1-9] | 3 [1-9] | 0.612 |
| Acute Respiratory Distress Syndrome | 70 (18.3) | 59 (19.9) | 11 (12.8) | 0.181 | 69.0 (20.0) | 7.8 (10.5) | 0.042 |
| Vasopressors use | 316 (82.5) | 242 (81.5) | 74 (86.0) | 0.412 | 239.6 (81.4) | 62.8 (85.3) | 0.435 |
| Vasopressors duration *^f^* | 2 [1-4] | 2 [1-4] | 2 [1-5] | 0.556 | 2 [1-4] | 2 [1-4] | 0.700 |
| Acute kidney injury | 268 (70.0) | 208 (70.0) | 60 (69.8) | 1.000 | 206.2 (70.1) | 49.9 (67.8) | 0.700 |
| AKI KDIGO stage *^g^* |  |  |  | 0.474 |  |  | 0.816 |
| *Stage I* | 53 (19.5) | 38 (17.9) | 15 (25.0) |  | 39.0 (18.5) | 11.1 (22.3) |  |
| *Stage II* | 59 (21.7) | 47 (22.2) | 12 (20.0) |  | 46.7 (22.2) | 10.1 (20.3) |  |
| *Stage III* | 160 (58.8) | 127 (59.9) | 33 (55.0) |  | 124.7 (59.2) | 28.6 (57.4) |  |
| Renal Replacement Therapy | 83 (21.7) | 64 (21.5) | 19 (22.1) | 1.000 | 64.2 (21.2) | 14.9 (20.2) | 0.850 |
| Antibiotics duration*^h^* | 7 [5-10] | 7 [5-9] | 7 [5-11] | 0.296 | 7 [5-9] | 7 [5-10] | 0.478 |
| **Outcomes** |  |  |  |  |  |  |  |
| ICU length of stay | 7 [3-17] | 7 [3-17] | 8 [3-17] | 0.471 | 7 [3-17] | 8 [3-17] | 0.601 |
| Hospital length of stay*^i^* | 27 [14-49] | 25 [13-48] | 29 [16-50] | 0.497 | 25 [13-48] | 27 [16-49] | 0.763 |
| ICU mortality | 108 (28.2) | 78 (26.3) | 30 (34.9) | 0.153 | 77.6 (26.4) | 23.5 (31.9) | 0.332 |
| In-hospital mortality | 128 (33.4) | 94 (31.6) | 34 (39.5) | 0.217 | 94.1 (32.0) | 26.4 (35.9) | 0.518 |

Data are presented as median [Q1-Q3] and n (%).

Abbreviations: AKI: Acute Kidney Injury, KDIGO: Kidney Disease Improving Global Outcomes, ICU: Intensive Care Unit

Missing values: ^a^ n=1; ^b^ n=61, ^c^ n=43, ^d^ n=42, ^e^ n=76, ^i^ n=58, ^g^ n=112, ^h^ n=9, ^i^ n=19

**Supplementary Table 5.** Risk factors for *Candida* spp*.* isolation (backward stepwise-based model)

| **Variables** | Unadjusted analysis | | |  | Adjusted analysis*^a^* | | |
| --- | --- | --- | --- | --- | --- | --- | --- |
|  | OR | 95%CI | *p* value |  | aOR | 95%CI | *p* value |
| **Baseline features** |  |  |  |  |  |  |  |
| Age (years) | 0.99 | 0.97-1.01 | 0.447 |  |  |  |  |
| Female sex | 1.14 | 0.68-1.89 | 0.611 |  |  |  |  |
| **Comorbidities** |  |  |  |  |  |  |  |
| Obesity (BMI>30) | 0.70 | 0.38-1.24 | 0.231 |  |  |  |  |
| Malnutrition | 1.80 | 1.09-3.02 | 0.023 |  | 1.84 | 1.10-3.14 | 0.022 |
| Cirrhosis | 0.66 | 0.22-1.63 | 0.406 |  |  |  |  |
| Diabetes | 1.35 | 0.75-2.37 | 0.298 |  |  |  |  |
| Chronic kidney failure | 0.16 | 0.01-0.80 | 0.080 |  |  |  |  |
| Congestive heart failure | 1.40 | 0.64-2.86 | 0.373 |  |  |  |  |
| Any immunosuppression | 1.00 | 0.61-1.64 | 0.986 |  |  |  |  |
| Solid organ transplantation | 0.45 | 0.02-2.51 | 0.457 |  |  |  |  |
| Solid tumor | 1.07 | 0.62-1.80 | 0.801 |  |  |  |  |
| Hematological malignancy | 0.60 | 0.14-1.82 | 0.417 |  |  |  |  |
| Long term corticosteroids | 0.61 | 0.20-1.50 | 0.321 |  |  |  |  |
| Antimicrobial therapy within the last 3 months | 2.21 | 1.25-3.84 | 0.006 |  | 2.15 | 1.20-3.81 | 0.009 |
| **Features of intra-abdominal infection** |  |  |  |  |  |  |  |
| Setting of infection acquisition |  |  |  |  |  |  |  |
| ‘Community acquired’ peritonitis | Ref | ref | ref |  |  |  |  |
| ‘Early-onset nosocomial’ peritonitis (<7 days) | 0.84 | 0.41-1.62 | 0.610 |  |  |  |  |
| ‘Late onset nosocomial’ peritonitis (>7 days) | 1.80 | 1.03-3.15 | 0.037 |  |  |  |  |
| Postoperative peritonitis | 1.51 | 0.91-2.48 | 0.107 |  |  |  |  |
| Upper GI tract source | 1.81 | 1.09-2.99 | 0.021 |  | 2.04 | 1.20-3.44 | 0.008 |
| Diffuse peritonitis | 1.64 | 0.97-2.88 | 0.071 |  | 1.75 | 1.01-3.13 | 0.049 |
| **Initial severity and management** |  |  |  |  |  |  |  |
| Time from hospital admission and peritonitis (days) | 1.00 | 0.99-1.02 | 0.511 |  |  |  |  |
| Sepsis at ICU admission | 1.04 | 0.60-1.76 | 0.876 |  |  |  |  |
| Septic shock at ICU admission | 1.08 | 0.66-1.77 | 0.761 |  |  |  |  |
| SAPS II | 1.00 | 0.98-1.01 | 0.893 |  |  |  |  |
| Timing of source control intervention |  |  |  |  |  |  |  |
| ‘Emergency’ (<2H) | ref. | ref. | ref. |  |  |  |  |
| ‘Urgent’ (2-6H) | 1.14 | 0.56-2.42 | 0.730 |  |  |  |  |
| ‘Delayed ‘ (>6H) | 1.34 | 0.65-2.92 | 0.437 |  |  |  |  |

Data are presented as Odds Ratio (OR) and 95% CIs.

Abbreviations: GI: Gastro-Intestinal; ICU: Intensive Care Unit; SAPS II: Simplified Acute Physiology Score II

*^a^*Multivariable logistic regression with backward stepwise selection (entry p<0.2, retention p<0.05) was performed, selecting following variables: Malnutrition, Antimicrobial therapy within the last 3 months, Upper GI tract source and diffuse peritonitis

**Supplementary Table 6.** Risk factors for *Candida* spp*.* isolation, including patients with candidemia

| **Variables** | Unadjusted analysis | | |  | Adjusted analysis*^a^* | | |
| --- | --- | --- | --- | --- | --- | --- | --- |
|  | OR | 95%CI | *p* value |  | aOR | 95%CI | *p* value |
| **Baseline features** |  |  |  |  |  |  |  |
| Age (years) | 0.99 | 0.97-1.01 | 0.394 |  |  |  |  |
| Female sex | 1.15 | 0.69-1.89 | 0.582 |  |  |  |  |
| **Comorbidities** |  |  |  |  |  |  |  |
| Obesity (BMI>30) | 0.65 | 0.35-1.14 | 0.146 |  |  |  |  |
| Malnutrition | 1.79 | 1.09-2.96 | 0.022 |  | 1.88 | 1.14-3.16 | 0.015 |
| Cirrhosis | 0.62 | 0.20-1.59 | 0.337 |  |  |  |  |
| Diabetes | 1.33 | 0.75-2.30 | 0.319 |  |  |  |  |
| Chronic kidney failure | 0.15 | 0.01-0.76 | 0.070 |  |  |  |  |
| Congestive heart failure | 1.31 | 0.61-2.66 | 0.478 |  |  |  |  |
| Any immunosuppression | 1.09 | 0.67-1.76 | 0.739 |  |  |  |  |
| Solid organ transplantation | 0.43 | 0.02-2.36 | 0.422 |  |  |  |  |
| Solid tumor | 1.09 | 0.65-1.82 | 0.735 |  |  |  |  |
| Hematological malignancy | 0.76 | 0.21-2.09 | 0.621 |  |  |  |  |
| Long term corticosteroids | 0.69 | 0.25-1.62 | 0.432 |  |  |  |  |
| Antimicrobial therapy within the last 3 months | 2.26 | 1.30-3.89 | 0.004 |  | 2.17 | 1.23-3.79 | 0.006 |
| **Features of intra-abdominal infection** |  |  |  |  |  |  |  |
| Setting of infection acquisition |  |  |  |  |  |  |  |
| ‘Community acquired’ peritonitis | ref | ref | ref |  |  |  |  |
| ‘Early-onset nosocomial’ peritonitis (<7 days) | 0.96 | 0.49-1.81 | 0.897 |  |  |  |  |
| ‘Late onset nosocomial’ peritonitis (>7 days) | 1.98 | 1.14-3.42 | 0.014 |  |  |  |  |
| Postoperative peritonitis | 1.64 | 1.00-2.66 | 0.047 |  |  |  |  |
| Upper GI tract source | 1.85 | 1.13-3.03 | 0.014 |  | 2.44 | 1.19-3.28 | 0.008 |
| Diffuse peritonitis | 1.40 | 0.84-2.37 | 0.204 |  |  |  |  |
| **Initial severity and management** |  |  |  |  |  |  |  |
| Time from hospital admission and peritonitis (days) | 1.00 | 0.99-1.02 | 0.454 |  |  |  |  |
| Sepsis at ICU admission | 1.13 | 0.67-1.88 | 0.646 |  |  |  |  |
| Septic shock at ICU admission | 1.06 | 0.65-1.71 | 0.824 |  |  |  |  |
| SAPS II | 1.00 | 0.99-1.01 | 0.969 |  |  |  |  |
| Timing of source control intervention |  |  |  |  |  |  |  |
| ‘Emergency’ (<2H) | ref. | ref. | ref. |  |  |  |  |
| ‘Urgent’ (2-6H) | 1.45 | 0.70-3.26 | 0.337 |  |  |  |  |
| ‘Delayed ‘ (>6H) | 1.75 | 0.83-3.98 | 0.159 |  |  |  |  |

Data are presented as Odds Ratio (OR) and 95% CIs.

Abbreviations: GI: Gastro-Intestinal; ICU: Intensive Care Unit; SAPS II: Simplified Acute Physiology Score II

*^a^*Multivariable logistic regression with backward stepwise selection (entry p<0.2, retention p<0.05) was performed, selecting following variables: Malnutrition, Antimicrobial therapy within the last 3 month and Upper GI tract source

**Supplementary Table 7.** Clinical course of patients with *Candida* spp*.* isolation according to early antifungal therapy (raw and weighted population)

|  | *Raw population* | | | | *Weighted population* | | | |
| --- | --- | --- | --- | --- | --- | --- | --- | --- |
|  | **All patients**  **n=81** | **No early antifungal therapy**  **n=50** | **Early antifungal therapy**  **n=31** | ***p* value** |  | **No early antifungal therapy**  **n=42.12** | **Early antifungal therapy**  **n=22.09** | ***p* value** |
| **Antifungal therapy** |  |  |  |  |  |  |  |  |
| Antifungal therapy duration | 8 [6-14] | 8 [0-13] | 7 [2-10] | 0.257 |  | 8 [6-14] | 8 [2-11] | 0.586 |
| **Source control achievement** |  |  |  |  |  |  |  |  |
| Successful at day 7 | 40 (49.4) | 23 (46.0) | 17 (54.8) | 0.586 |  | 19.4 (46.1) | 12.6 (57.1) | 0.406 |
| Failure at day 7, persistent inflammation^a^ | 36 (55.4) | 26 (61.9) | 10 (43.5) | 0.243 |  | 24.1 (67.8) | 5.7 (34.4) | 0.018 |
| Failure at day 7, additional radiological intervention^b^ | 7 (10.0) | 5 (11.4) | 2 (7.7) | 0.934 |  | 3.4 (9.1) | 0.9 (5.0) | 0.463 |
| Failure at day 7, additional surgical intervention^c^ | 24 (29.6) | 15 (34.1) | 4 (15.4) | 0.155 |  | 14.1 (37.9) | 2.6 (14.7) | 0.072 |
| Additional intervention required within 28 days after peritonitis onset | 24 (29.6) | 18 (36.0) | 6 (19.4) | 0.179 |  | 16.0 (38.1) | 3.5 (16.0) | 0.054 |
| **Organ dysfunctions and life-sustaining therapies** |  |  |  |  |  |  |  |  |
| Mechanical ventilation | 63 (77.8) | 38 (76.0) | 25 (80.6) | 0.831 |  | 31.2 (73.9) | 19.0 (86.0) | 0.196 |
| Mechanical ventilation duration^d^ | 3 [1-8] | 3 [1-7] | 2 [1-8] | 0.878 |  | 3 [1-7] | 2 [1-9] | 0.717 |
| Acute Respiratory Distress Syndrome | 10 (12.3) | 7 (14.0) | 3 (9.7) | 0.820 |  | 6.2 (14.8) | 3.4 (15.3) | 0.967 |
| Vasopressors | 69 (85.2) | 43 (86.0) | 26 (83.9) | 1.000 |  | 35.6 (84.6) | 18.5 (83.9) | 0.942 |
| Vasopressors duration^e^ | 2 [1-5] | 2 [1-3] | 3 [2-6] | 0.102 |  | 2 [1-3] | 3 [2-6] | 0.039 |
| Acute kidney injury | 55 (67.9) | 34 (68.0) | 21 (67.7) | 1.000 |  | 27.8 (65.9) | 16.4 (74.4) | 0.459 |
| AKI KDIGO stage ^f^ |  |  |  | 0.052 |  |  |  | 0.083 |
| Stage I | 14 (25.5) | 7 (20.6) | 7 (33.3) |  |  | 5.8 (20.8) | 5.7 (34.9) |  |
| Stage II | 12 (21.8) | 11 (32.4) | 1 (4.8) |  |  | 8.0 (28.8) | 0.6 (3.5) |  |
| Stage III | 29 (52.7) | 16 (47.1) | 13 (61.9) |  |  | 14.0 (50.4) | 10.1 (61.6) |  |
| Renal Replacement Therapy | 19 (23.5) | 8 (16.0) | 11 (35.5) | 0.082 |  | 7.2 (17.2) | 8.4 (38.1) | 0.081 |
| **Outcomes** |  |  |  |  |  |  |  |  |
| ICU length of stay | 8 [3-16] | 7 [3-18] | 8 [3-14] | 0.800 |  | 6 [3-14] | 8 [3-16] | 0.673 |
| Hospital length of stay^g^ | 27 [16-50] | 30 [18-52] | 21 [9-36] | 0.053 |  | 27 [16-51] | 24 [9-50] | 0.536 |
| ICU mortality | 28 (34.6) | 16 (32.0) | 12 (38.7) | 0.706 |  | 13.6 (32.2) | 7.9 (35.5) | 0.788 |
| In-hospital mortality | 31 (38.3) | 18 (36.0) | 13 (41.9) | 0.765 |  | 14.9 (35.4) | 8.3 (37.8) | 0.851 |

Data are presented as median [Q1-Q3] and n (%).

Abbreviations: AKI: Acute Kidney Injury, ICU: Intensive care Unit, KDIGO: Kidney Disease Improving Global Outcomes

Missing values: ^a^ n=16, ^b^ n=11, ^c^ n=11, ^d^ n=15, ^e^ n=15, ^f^ n=26, *^g^* n=4

**Supplementary Table 8.** Baseline characteristics of patients with *Candida* spp. isolation according to early antifungal therapy, including patients with candidemia (raw and weighted population)

|  | *Raw population* | | | | | *Weighted population* | | | |
| --- | --- | --- | --- | --- | --- | --- | --- | --- | --- |
|  | **All patients**  **n=86** | **No early antifungal therapy**  **n=52** | **Early antifungal therapy**  **n=34** | ***p* value** | ***SMD*** | **No early antifungal therapy**  **n=42.12** | **Early antifungal therapy**  **n=23.84** | ***p* value** | **SMD** |
| **Baseline features** |  |  |  |  |  |  |  |  |  |
| Age (years) | 68 [58-74] | 69 [59-75] | 66 [56-72] | 0.342 | 0.250 | 68 [58-73] | 70 [59-73] | 0.990 | 0.081 |
| Female sex | 32 (37.2) | 16 (30.8) | 16 (47.1) | 0.194 | 0.162 | 14.8 (36.3) | 10.9 (45.7) | 0.390 | 0.106 |
| **Comorbidities** |  |  |  |  |  |  |  |  |  |
| Obesity (BMI>30) | 17 (19.8) | 8 (15.4) | 9 (26.5) | 0.324 | 0.111 | 6.6 (15.8) | 4.6 (9.2) | 0.687 | 0.034 |
| Malnutrition | 55 (64.0) | 33 (63.5) | 22 (64.7) | 1.000 | 0.012 | 25.3 (60.1) | 14.8 (62.0) | 0.876 | 0.019 |
| Diabetes | 22 (25.6) | 9 (17.3) | 13 (38.2) | 0.055 | 0.209 | 8.0 (19.1) | 6.8 (28.4) | 0.342 | 0.094 |
| Any immunosuppression | 42 (48.8) | 24 (46.2) | 18 (52.9) | 0.693 | 0.067 | 18.9 (44.9) | 12.0 (50.2) | 0.668 | 0.053 |
| Solid tumor | 28 (32.6) | 18 (34.6) | 10 (29.4) | 0.789 | 0.052 | 14.0 (33.3) | 7.8 (32.5) | 0.949 | 0.008 |
| Antimicrobial therapy within the last 3 months | 27 (31.4) | 16 (30.8) | 11 (32.4) | 1.000 | 0.016 | 13.5 (32.1) | 8.8 (36.8) | 0.699 | 0.047 |
| **Features of intra-abdominal infection** |  |  |  |  |  |  |  |  |  |
| Setting of infection acquisition |  |  |  | 0.034 |  |  |  | 0.632 |  |
| ‘Community-acquired’ peritonitis | 36 (41.9) | 16 (30.8) | 20 (58.8) |  | 0.281 | 15.4 (36.6) | 11.6 (48.8) |  | 0.123 |
| ‘Early-onset nosocomial’ peritonitis (<7 days) | 16 (18.6) | 12 (23.1) | 4 (11.8) |  | 0.113 | 8.9 (21.1) | 3.8 (16.0) |  | 0.051 |
| ‘Late-onset nosocomial’ peritonitis (>7 days) | 34 (39.5) | 24 (46.2) | 10 (29.4) |  | 0.167 | 17.8 (42.4) | 8.4 (35.2) |  | 0.072 |
| Postoperative peritonitis | 40 (46.5) | 27 (51.9) | 13 (38.2) | 0.306 | 0.137 | 21.1 (50.0) | 10.1 (42.4) | 0.545 | 0.076 |
| Source of peritonitis: Upper GI tract^a^ | 37 (44.6) | 16 (32.7) | 21 (61.8) | 0.016 | 0.272 | 15.0 (35.6) | 12.3 (51.6) | 0.202 | 0.159 |
| Diffuse peritonitis^b^ | 59 (72.0) | 34 (70.8) | 25 (73.5) | 0.985 | 0.024 | 30.0 (71.3) | 17.2 (72.1) | 0.944 | 0.008 |
| **Initial severity and management** |  |  |  |  |  |  |  |  |  |
| Time from hospital admission and peritonitis (days) | 4 [0-13] | 6 [1-13] | 0 [0-10] | 0.072 | 0.038 | 4 [0-13] | 2 [0-11] | 0.477 | 0.007 |
| Sepsis at ICU admission | 28 (32.6) | 19 (36.5) | 9 (26.5) | 0.460 | 0.101 | 16.0 (38.1) | 8.1 (34.1) | 0.753 | 0.040 |
| Septic shock at ICU admission | 42 (48.8) | 21 (40.4) | 21 (61.8) | 0.086 | 0.213 | 17.0 (40.4) | 11.8 (49.6) | 0.454 | 0.093 |
| SAPS II^c^ | 50 [40-62] | 51 [38-61] | 50 [42-60] | 0.840 | 0.041 | 50 [33-60] | 49 [39-58] | 0.765 | 0.052 |
| Timing of source control intervention^d^ |  |  |  | 0.125 |  |  |  | 0.703 |  |
| ‘Emergency’ (<2H)’ | 11 (12.8) | 3 (6.5) | 7 (21.2) |  | 0.110 | 5.9 (13.9) | 5.3 (2.1) |  | 0.081 |
| ‘Urgent’ (2-6H) | 42 (48.8) | 23 (50.0) | 16 (48.5) |  | 0.000 | 19.2 (5.6) | 9.7 (40.7) |  | 0.049 |
| ‘Delayed’ (>6H) | 33 (38.4) | 20 (43.4) | 10 (30.3) |  | 0.110 | 17.1 (40.5) | 8.9 (37.3) |  | 0.032 |
| **Microbiology findings** |  |  |  |  |  |  |  |  |  |
| *Candida* spp. isolation^e^ |  |  |  | 0.434 |  |  |  | 0.532 |  |
| Peritoneal fluid only | 79 (94.0) | 48 (96.0) | 31 (91.2) |  | 0.050 | 40.3 (95.6) | 22.1 (92.5) |  | 0.031 |
| Blood culture only | 4 (4.8) | 2 (4.0) | 2 (5.9) |  | 0.020 | 1.9 (4.4) | 1.4 (5.8) |  | 0.014 |
| Peritoneal fluid and blood culture | 1 (1.2) | 0 (0.0) | 1 (2.9) |  | 0.029 | 0.0 (0.0) | 0.4 (1.7) |  | 0.017 |
| Yeast-positive direct examination of peritoneal fluid | 17 (19.8) | 10 (19.2) | 7 (20.6) | 1.000 | 0.014 | 8.9 (21.0) | 5.5 (23.3) | 0.835 | 0.022 |
| *Candida albicans* | 64 (74.4) | 41 (78.8) | 23 (67.6) | 0.362 | 0.112 | 32.8 (77.9) | 17.5 (73.6) | 0.670 | 0.043 |
| Pure *Candida* spp. culture | 19 (22.1) | 8 (15.4) | 11 (32.4) | 0.112 | 0.146 | 6.6 (15.7) | 5.6 (23.5) | 0.374 | 0.079 |
| Fluconazole-resistant isolate^f^ | 8 (10.4) | 5 (10.9) | 3 (9.7) | 0.434 | 0.008 | 4.1 (9.7) | 1.6 (6.5) | 0.571 | 0.032 |

Data are presented as median [Q1-Q3] and n (%).

Abbreviations: BMI: Body Mass Index, GI: Gastro-intestinal, ICU: Intensive Care Unit; SAPSII: Simplified Acute Physiology Score II

Missing values: ^a^ n=3; ^b^ n=4; ^c^ n=6; ^d^ n=7; ^e^ n=2; ^f^ n=9

**Supplementary Table 9.** Clinical course of patients with *Candida* spp*.* isolation according to early antifungal therapy, including patients with candidemia (raw and weighted population)

|  | *Raw population* | | | | *Weighted population* | | | |
| --- | --- | --- | --- | --- | --- | --- | --- | --- |
|  | **All patients**  **n=86** | **No early antifungal therapy**  **n=52** | **Early antifungal therapy**  **n=34** | ***p* value** |  | **No early antifungal therapy**  **n=42.12** | **Early antifungal therapy**  **n=23.84** | ***p* value** |
| **Antifungal therapy** |  |  |  |  |  |  |  |  |
| Antifungal therapy duration | 9 [6-14] | 9 [6-14] | 9 [5-11] | 0.179 |  | 8 [6-14] | 10 [6-14] | 0.787 |
| **Source control achievement** |  |  |  |  |  |  |  |  |
| Successful at day 7 | 41 (47.7) | 24 (46.2) | 17 (50.0) | 0.898 |  | 19.2 (45.5) | 12.7 (53.4) | 0.527 |
| Failure at day 7, persistent inflammation^a^ | 41 (58.6) | 28 (63.6) | 13 (50.0) | 0.563 |  | 24.1 (67.0) | 8.3 (43.1) | 0.081 |
| Failure at day 7, additional radiological intervention^b^ | 7 (9.3) | 5 (10.9) | 2 (6.9) | 0.866 |  | 3.3 (8.7) | 0.9 (4.5) | 0.411 |
| Failure at day 7, additional surgical intervention^c^ | 22 (29.3) | 16 (34.8) | 6 (20.7) | 0.296 |  | 14.0 (3./2) | 4.6 (22.7) | 0.270 |
| Additional intervention required within 28 days after peritonitis onset | 27 (31.4) | 19 (36.5) | 8 (23.5) | 0.301 |  | 15.9 (37.8) | 5.6 (23.3) | 0.224 |
| **Organ dysfunctions and life-sustaining therapies** |  |  |  |  |  |  |  |  |
| Mechanical ventilation | 68 (79.1) | 40 (76.9) | 28 (82.4) | 0.738 |  | 31.2 (74.2) | 20.5 (85.8) | 0.219 |
| Mechanical ventilation duration^d^ | 3 [1-9] | 3 [1-8] | 2 [1-9] | 0.896 |  | 3 [1-8] | 3 [1-9] | 0.557 |
| Acute Respiratory Distress Syndrom | 11 (12.8) | 7 (13.5) | 4 (11.8) | 1.000 |  | 6.1 (14.5) | 4.2 (17.7) | 0.753 |
| Vasopressors use | 74 (86.0) | 45 (86.5) | 29 (85.3) | 1.000 |  | 36.3 (86.2) | 20.0 (83.8) | 0.786 |
| Vasopressors duration^e^ | 2 [1-5] | 2 [1-3] | 3 [2-6] | 0.059 |  | 2 [1-3] | 4 [2-7] | 0.013 |
| Acute kidney injury | 60 (69.8) | 36 (69.2) | 24 (70.6) | 1.000 |  | 28.5 (67.7) | 18.0 (75.3) | 0.480 |
| AKI KDIGO stage ^f^ |  |  |  | 0.038 |  |  |  | 0.059 |
| Stage I | 15 (25.0) | 7 (19.4) | 8 (33.3) |  |  | 5.5 (19.1) | 6.7 (37.0) |  |
| Stage II | 12 (20.0) | 11 (30.6) | 1 (4.2) |  |  | 7.7 (27.0) | 0.6 (3.4) |  |
| Stage III | 33 (55.0) | 18 (50.0) | 15 (62.5) |  |  | 15.4 (53.8) | 10.7 (59.5) |  |
| Renal Replacement Therapy | 19 (22.1) | 8 (15.4) | 11 (32.4) | 0.112 |  | 7.0 (16.6) | 7.6 (31.7) | 0.155 |
| **Outcomes** |  |  |  |  |  |  |  |  |
| ICU length of stay | 8 [3-17] | 8 [4-18] | 8 [4-16] | 0.958 |  | 6 [3-16] | 8 [3-16] | 0.446 |
| Hospital length of stay^g^ | 29 [16-50] | 30 [18-54] | 24 [9-40] | 0.111 |  | 27 [17-51] | 28 [10-45] | 0.634 |
| ICU mortality | 30 (34.9) | 16 (30.3) | 14 (42.4) | 0.355 |  | 13.2 (31.3) | 8.7 (36.5) | 0.647 |
| In-hospital mortality | 34 (39.5) | 19 (35.8) | 15 (45.5) | 0.510 |  | 15.8 (37.4) | 9.6 (40.4) | 0.550 |

Data are presented as median (IQR: interquartiles), n (%).

Abbreviations: AKI: Acute Kidney Injury, ICU: Intensive care Unit, KDIGO: Kidney Disease Improving Global Outcomes

Missing values: ^a^ n=16, ^b^ n=11, ^c^ n=11, ^d^ n=15, ^e^ n=16, ^f^ n=26, *^g^* n=4
